# Comparative quality assessment of traditional vs. improved dried Bombay duck (*Harpodon nehereus*) under different storage conditions: Solar chimney dryer a low‐cost improved approach for nutritional dried fish

**DOI:** 10.1002/fsn3.2631

**Published:** 2021-10-17

**Authors:** Biplob Dey Mithun, Md. Sazedul Hoque, Martin Louis Van Brakel, Md. Mahmudul Hasan, Shaida Akter, Mohd. Rezaul Islam

**Affiliations:** ^1^ Department of Fisheries Technology Patuakhali Science and Technology University Dumki, Patuakhali Bangladesh; ^2^ Independent Consultant Papendrecht The Netherlands; ^3^ Department of Fisheries Management Bangabandhu Sheikh Mujibur Rahman Agricultural University Salna Gazipur Bangladesh; ^4^ Associate Researcher Bangladesh Aquaculture‐Horticulture for Nutrition Collaborative Research Program WorldFish‐Bangladesh, SS View Barisal Bangladesh; ^5^ Bangladesh Fisheries Research Institute Freshwater Station Saidpur, Nilphamari Bangladesh

**Keywords:** bombay duck, dried fish, quality analysis, solar chimney dryer, storage condition

## Abstract

Dried fish and fishery products provide important and affordable dietary sources of animal protein. The present study compared the sensory, microbial, and biochemical quality of bombay duck (*Harpadon nehereous*) dried fish produced using improved versus traditional open‐air drying method. The sensory, microbial, and biochemical quality was determined following quality index, total plate count (TPC), and Association of Official Analytical Chemists (AOAC) method, respectively. The sensory quality results indicate highly acceptable dried fish with the improved method compared to traditional method at both initial and storage condition. The microbial load (APC, TEC, TViC, and TSC) of improved dried fish was within internationally permissible microbiological standards for food whereas traditionally dried fish exceeded the permissible limit. The biochemical composition (protein, lipid, ash, and moisture) of improved dried fish had better nutritional value than the traditional dried fish. After 60 days storage time, samples packaged under HDPE conditions exhibited excellent organoleptic characteristics, lower microbial load, and lower biochemical decomposition than samples packaged in LDPE. The above results confirmed that the solar chimney dryer produced superior quality of dried fish compared to the traditional open‐air drying method both initially and after storage, while packaging dried fish under HDPE conditions extended shelf life further.

## Graphical abstract

Solar chimney dryer produces premium quality dried fish at low cost. Sensory, microbial, and biochemical quality of improved dried fish is superior to the traditional one. HDPE packaged dried fish retained better sensory, microbial, and biochemical quality than LDPE after 60 days of storage.

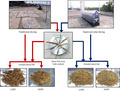

## 1 INTRODUCTION

Fisheries and aquatic resources contributed 3.50% to the national gross domestic production (GDP) and 25.71% to the agricultural GDP, 1.5% of total export earnings, 60% of animal protein supply, and generated employment to more than 11% population in Bangladesh (DoF, 2019). Among the different fish and fishery products, dried fish has significant relevance to the socio‐economic development and nutrition security of the nation (Felicia & Jamila, 2003). A number of fish species such as Bombay duck (*Harpodon nehereus*), pomfret (*Stromateus chinensis*, *Parastromateus niger* and *Stromateus cinereus*), jewfish (*Johnius argenteus*, *Otolithoides argenteus*, *Johnius diacantus* and *Otolithoides brunnes*), ribbon fish (*Trichiurus haumela*), anchovy (*Setipina taty*), and shrimp (*Penaeus* spp.) are used for commercial production of dried fish during October to March in the coastal districts of Bangladesh (Amin et al., 2012; Nowsad, 2007).

Sun drying is a traditional method for preservation of fish and other perishable foods which is commonly employed in any developing countries around the world (Lamidi et al., 2019). Solar drying technologies do have considerable potential for application in small‐scale food processing units in rural areas, as alternative to expensive mechanical drying technologies under controlled conditions. Existing commercial fish drying in Bangladesh mostly involves drying in the open air, making use of energy from sunlight without any mechanical processing (Balachandran, 2001). Traditionally, sun drying is done directly on sand at the sea beach and/or on mats woven from hogla (*Typha angustata*) leaves without or with minimum washing and gutting of raw materials (Nowsad, 2007).

In view of public health and food safety aspects, the quality of fish and fishery products is a major concern for local and global consumers (Huss et al., 2003). Traditionally dried fish is associated with hygiene, quality, and food safety (chemical contamination) problems arising at different steps along the supply chain from preparation to consumption (Begum et al., 2017). Overall, low quality or spoiled raw materials, lack of personal hygiene, use of harmful chemicals, and improper processing, packaging, and storage conditions are the major constraints for quality management, resulting as a consequence in low market value of dried fish (Majumdar et al., 2017; Nowsad, 2007). Long and uncontrolled drying times and nonuniform exposure to temperature and sun leave the product exposed to contamination, resulting in a low quality of dried products. Commercial processors often apply several harmful insecticides (nogos, nuvacorn, endrin, malathion, dichloro diphenyl trichloroethane, and basudin) in dried fish to prevent insect infestation and microbial contaminations during drying and storage which are hazardous for human health (Begum et al., 2017).

Several studies have been conducted on sensory, nutritional, and microbial quality aspects, of sun‐dried Bombay duck from coastal area of Bangladesh (Jamil et al., 2017; Paul et al., 2018; Pravakar et al., 2013; Rasul et al., 2020; Siddique & Aktar, 2011). The physical and organoleptic qualities of most of the traditional sun‐dried products in Bangladesh are not attractive to the consumer (Hasan et al., 2006). Microbial contamination is another major cause of spoilage and deterioration of nutritional quality of dried fish. Among the biochemical constituents (moisture, crude protein, lipid, and ash) of dried fish, moisture content has the largest impact on its nutritive value and microbial load. Even properly dried fish deteriorates if it is not well packaged and stored. Effective packaging and storage reduce oxidation, rehydration, microbial, and biochemical spoilage and protect the product from physical damage and nutritional losses (Omodara, 2015). As a low‐cost and improved technology, the UC Davis solar chimney dryer is designed to provide efficient drying of food materials even in hazy or partially cloudy conditions, without external energy inputs other than direct sunlight. The design of the dryer is such that it creates a continuous airflow, which increases the speed of drying and rapidly removes moisture. The dryer can be constructed from locally available materials that reduce the dryer construction cost and drying time, while minimizing insect infestation and pollution with dust (Horticulture Innovation Lab, 2019).

Considering the above factors, the purpose of the present study was to compare the sensory, microbial, and biochemical quality and nutritional properties of fish dried using an improved low‐cost solar drying technology, the UC Davis solar chimney dryer, against quality and nutritional properties of traditional open‐air dried fish, under different packaging conditions and storage periods.

## 2 MATERIALS AND METHODS

### 2.1 UC Davis solar chimney dryer construction

A full‐sized dryer up to 12 feet long, consisting of three components, namely chimney, drying table, and trays, was constructed according to specifications in the construction manual (Horticulture Innovation Lab, 2019) provided online by the designer. First, the chimney was made up of a vertical frame of four tall poles, raising at full height about 6 ft (2 m) above the top of the drying bed. The width of the chimney, here 2 ft wide, should be equal to the width of the drying table and trays. The chimney was lined with clear polythene plastic sheet, and the top opening was covered with nylon mesh. The chimney was anchored to the ground, by digging in the poles to stabilize the structure.

**FIGURE 1:**
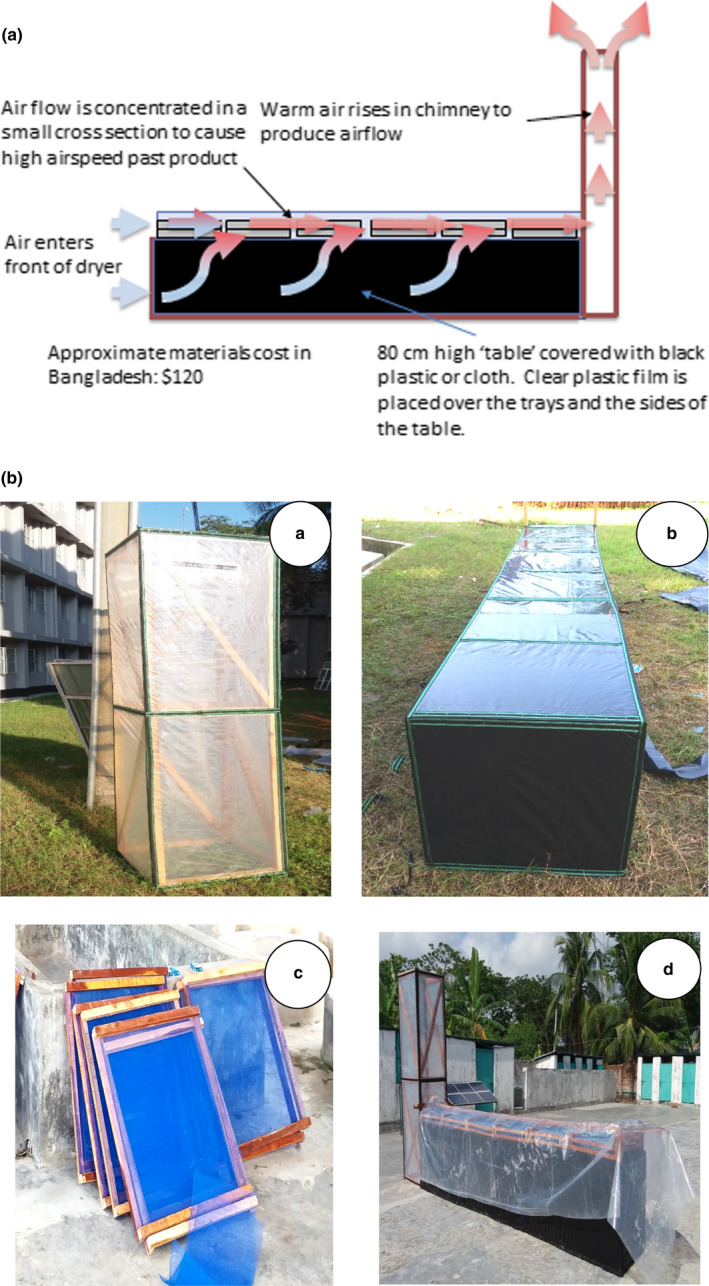
(a) Construction design and working principle of UC Davis Solar Chimney Dryer. (b) Different parts of UC Davis Chimney Dryer; (a) chimney; (b) table; (c) tray; and (d) assembled dryer are presented during drying

Second, the drying table, measuring 12 ft long and 2 ft wide, was constructed by stretching black plastic or dark fabric tightly over the top, sides, and ends of the table frame and stapled into place. Third, the trays were made using four (4) strips of wood, each measuring 2 ft in length. The floor of the trays consisted of iron mesh, cut in squares 2x2 ft length x width, and stapled to two strips, pulled apart tightening the mesh, and then secured by the other two strips (Figure 1a,b). The trays can be stacked in layers, up to three depending on the volume of raw materials to be dried. The table was then placed in horizontal position and connected carefully to the chimney in order to prevent any air leaks through the seals. At the junction of table and chimney, a window measuring 2 ft wide and 4 inches high was cut out from the clear polythene to create the inlet of airflow into the chimney. The raw fish were loaded on the trays and placed on the table structure aligning the window. Finally, the drying table (supporting the trays) was covered and sealed with clear polythene sheet, keeping the table open at the front for the intake of the incoming airflow. This way, a convection flow of solar‐heated air is created over the drying table, drawing moisture out of the product. The moist air is emitted through the chimney. The temperature of the airflow through the UC Davis solar chimney dryer was monitored at the middle trays on table covered with transparent polythene, by using a digital thermometer (AMF026, Qingdao, Shandong province, China). Fish was dried daily from 9 a.m. to 5 p.m. Temperature monitoring was conducted at 10 a.m., 1 p.m., and 4 p.m. both inside and outside of UC Davis solar chimney dryer. The results of the temperature reading are shown in Table 1.

**TABLE 1:** Daily temperature monitoring in inside and outside of UC Davis solar chimney dryer at three different times

Drying time & Temp.^a^	10 a.m.			1 p.m.			4 p.m.		
	Inside Dryer (°C)	Outside Dryer (°C)	DF^b^ (°C)	Inside Dryer (°C)	Outside Dryer (°C)	DF (°C)	Inside Dryer (°C)	Outside Dryer (°C)	DF (°C)
Day 1	33.36	28.13	5.23	37.42	30.16	7.26	37.33	30.11	7.22
Day 2	35.56	29.23	6.33	39.22	31.56	7.66	39.78	32.34	7.44
Day 3	33.74	28.35	5.39	38.60	30.78	7.82	37.50	30.23	7.27
Average	34.22	28.57	5.56	38.41	30.83	7.58	38.20	30.89	7.31

^a^ Daily drying time followed for 9a.m.‐5p.m., total 8 hr, where temperature monitored at 10a.m., 1p.m., and 4p.m.

^b^ DF—Temperature differences between inside and outside dryer.

### 2.2 Collection and processing of raw material

For this study, samples of traditionally produced dried bombay duck (*Harpadon nehereus*) fish, varying from 16 to 20 cm in length and 50 to 60 fish/kg dry weight, were collected from a seasonal dried fish processing settlement named Ashar char bordering Nidrar char, Taltoli, Barguna district. For improved drying, fresh bombay duck (*Harpadon nehereus*) fish samples (16–20 cm and 10–13 pieces/kg wet weight) were collected from Kuakata, Patuakhali district, and packed on ice in a styrofoam box following standard cold chain handling procedures. The samples were then transported by road to the laboratory in Patuakhali Science and Technology University (PSTU) within 3 hr. The collected bombay duck fish samples were washed, gutted, beheaded, and added with 5%–8% NaCl salt to be prepared for drying following the method described by Nowsad (2007). The prepared (10kg) bombay duck fish were then placed onto trays and dried in the UC Davis chimney dryer. After three days (24 hr) direct exposure to sunlight, the produced dried fish was yield of 20% (2.0 kg) with the moisture content 15.10%. Samples of both traditional (500gm; 25–30 pieces) and UC Davis Chimney dryer produced dried fish were then packaged separately (500gm; 30–35 pieces) under airtight conditions in high density polyethylene‐HDPE bags (HDPE, 0.94g/cm3, Xinghua Xinfeng Plastic Co. Ltd, China) and low density polyethylene‐LDPE bags (LDPE, 0.91 g/cm^3^, Xinghua Xinfeng Plastic Co. Ltd, China) and then stored at room temperature in an insulated plastic box for further quality (sensory, microbial and biochemical) analysis.

### 2.3 Quality analysis

#### 2.3.1 Sensory quality assessment of traditional and improved dried fish

Sensory tests were carried out by 15 trained panelists (seven male and eight female), using the Quality Index method (Howgate et al., 1992) with some modification of defects characteristics related to dried fish (Table 2). Sensory quality of dried fish was calculated, and grading decisions were made based on defect points (Table 3) by using the following formula‐

$$\text{Average grade points}=\frac{\text{Total grade point}}{\text{Number of characteristics}}$$

**TABLE 2:** Organoleptic assessment of dried fish based on defect characteristics

Characteristics of dried fish	Defect characters	Defect point	S‐1	S‐2	S‐3	S‐4
Color	Whitish and shiny	1				
	Off white to yellowish	2				
	Shining yellowish to brownish	3				
	Blackish inner and dark brownish outside	4				
	Blackish to bleached	5				
Odor	Natural dried fishy odor	1				
	Bland odor	2				
	Slightly to moderate fishy	3				
	Decomposed, sour odor	4				
	Extremely decomposed sour and spoiled	5				
Texture	Firm, tender and flexible	1				
	Slightly to moderate Soft	2				
	Extremely soft and slightly juicy	3				
	Brittle near to broken	4				
	Broken, juicy and skin raptured	5				
Flavor	Natural and slight salty	1				
	Slight to moderate flavor	3				
	Strong and spoiled	5				
Insect Infestation	No infestation	1				
	Slightly to moderate infestation	3				
	Completely infested by flies and insects	5				
General appearance	Excellent	1				
	Good	2				
	Slight to moderate good	3				
	Bad	4				
	Very bad	5				
Over all acceptability	Highly acceptable	1				
	Slight to moderate acceptable	2				
	Slightly Unacceptable	3				
	Very Unacceptable	4				
	Rejected	5				
Total defect point						
Average defect point						

**TABLE 3:** Grading of dried fish acceptance

Grade	Defect point	Degree of acceptance
A	<2	Excellent, Highly acceptable
B	2 to <5	Good/ acceptable
C	5	Rejected

#### 2.3.2 Microbiological quality assessment of traditional and improved dried fish

The bacteriological study was conducted following the procedure of standard plate count (SPC) (Cappuccino & Sherman, 1992), and dried fish samples were prepared following method of ISO (1995). Total bacterial colony count was performed in the form of aerobic plate count (APC) on nutrient agar media. About one (1) gram of blended dried fish sample was mixed thoroughly with 9 ml of sterile 1.5% peptone water obtained at 1:10 dilution. Then, 1 ml of supernatant was taken from the centrifuge tube and a 10‐fold serial dilution of the sample was performed with 0.9% physiological saline. Aliquots of 0.1 ml of the serial dilutions were inoculated (triplicate) onto nutrient agar media for APC. The plates were incubated in the incubator (JSGI‐10T, JSR, Korea) in an inverted position at 37°C for 24–48 hr.

APC was calculated by using the following formula:

$$\text{cfu}/g\;=\frac{\text{No}.\;\text{of colonies on petridish}\;\times 10\times \;\text{dilution factor}\;\times \;\text{volume of total stock solution}}{\text{Weight of dried fish sample}}$$

For specific bacterial count, 0.1 ml of the stock solution onto eosin methylene blue (EMB), xylose lysine deoxycholate (XLD), and thiosulfate citrate bile salts sucrose (TCBS) agar media were used for total *E. coli* count (TEC), total *Salmonella* count (TSC), and total *Vibrio* count (TViC), respectively. After 48–72 hr incubation, the colony colors on respective selective media were used for identification. The colorless or pale pink colonies on XLD agar were identified as *Salmonella typhimurium and Salmonella abony*, purple colonies with black center on EMB agar were identified as *Escherichia coli,* and yellow and bluish green colonies on TCBS agar were identified as *Vibrio cholerae* and *Vibrio parahaemolyticus*.

TEC, TSC, and TViC were calculated by using following formula:

$$\text{cfu}/g\;=\frac{\text{No}.\;\text{of colonies on petridish}\;\times 10\times \;\text{volume of total stock solution}\;}{\text{Weight of dried fish sample}}$$

#### 2.3.3 Biochemical quality assessment of traditional and improved dried fish

Proximate composition (moisture, protein, ash, and lipid) was determined following the method of AOAC (2000).

### 2.4 Statistical analysis of experimental data

The obtained data were subjected to analysis of variance (ANOVA), and mean comparisons were carried out by Duncans’ multiple range test using SPSS package (SPSS 16.00 for windows, SPSS Inc., Chicago, IL, USA) software. Significant difference was defined at *p* < .05.

## 3 RESULTS AND DISCUSSION

### 3.1 UC Davis solar chimney dryer

The solar chimney dryer can be assembled from locally available construction materials according to the specifications mentioned in section 2.1, at a cost of 150–200 USD. The production capacity of a dryer of this size varies between 60 and 80 kg raw fish depending on species and size of fish to be dried. A higher capacity of this dryer in terms of volume production can be obtained by increasing the length of the drying table, thus increasing number of trays. Temperature monitoring results showed that the temperature of the airflow measured within the chimney dryer ranged between 5.56 and 7.58°C higher than atmospheric temperature outside the chimney dryer (Table 1). The faster flow of evaporated moist water drawn out through the chimney, generated through solar heating of the airflow, thus provides superior drying efficiency, reducing drying time by 12–20 hr compared to traditional sun drying. Nowsad (2007) reported that fish drying efficiency depends on relative humidity, velocity and temperature of air, and also the rate at which surface moisture is carried away.

The unique feature of the UC Davis solar chimney dryer, which distinguishes this dryer from other solar dryers including solar tunnel dryers commonly used in many developing countries, is the “chimney.” Tunnel dryers often use exhaust fans for ventilation and require an external power source. In contrast, the UC Davis solar chimney dryer is designed to be used without any external power source like solar panel. Its design combines a large heat‐collection area (the drying table) and with a chimney that ensuring continuous air flow around the product, thus increasing the speed of drying compared to other designs. The small cross‐sectional area through which the air flow is forced speeds drying by generating high air speeds past the product, ensuring high temperatures and rapid water removal (Horticulture Innovation Lab, 2019).

### 3.2 Sensory quality of traditional and improved dried fish

**FIGURE 2:**
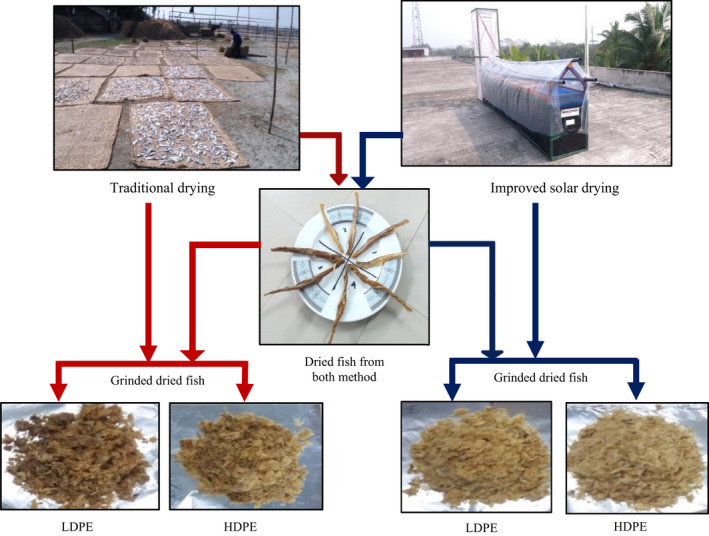
Differences in quality of dried fish produced from traditional and improved solar drying method under LDPE and HDPE packaging at 60 days storage time

In the present study, sensory quality of traditional and improved dried fish is compared and shown/reprent in Table 4 and Figure 2. The result indicated that the improved dried fish produced from UC Davis solar chimney dryer was rated “excellent” and highly acceptable by the panelists than the traditionally produced dried fish. The traditional dried fish scored high (2.42) on average defect point which is considered grade B, while the improved dried fish scored a low average defect point (1), considered grade A. The better sensory quality with higher acceptance of chimney dryer produced dried fish could be achieved by practice of maintaining proper sanitation and hygiene in processing. First, the structure of the dryer protects against contamination. The drying table with fish placed on trays is elevated from the ground and is covered with clear polythene sheet (Figure 1b). The traditional drying practice done in open air on sandy beach has a higher chance of dust and insect contamination. Second, differences in raw materials quality, preprocessing steps of washing, gutting, cleaning, salt ratio and quality, and utensils used resulted in the improved sensory quality of solar chimney dryer produced dried fish over traditionally dried fish. Third, the better theoretical and practical knowledge on hygiene and sanitations applied in improve drying in a trained or skilled manner by the researcher graduate student, whereas Nowsad (2007) stated that traditional processing mostly involved the unhygienic practice and unskilled/untrained manpower. The improved dried fish had a whitish color (Figure 2) and firm, tender texture, which was preferred by panelists due to its excellent and highly acceptable organoleptic characteristics. At the initial storage time (Day 0), traditional dried fish already displayed a blackish color and slight to moderately soft texture, which was rated as “moderately acceptable” for consumption by the panelists (Table 4, Figure 2). According to Pravakar et al. (2013), high quality dried bombay duck have a slightly silver to whitish color, while Huque et al. (2013) report whitish to yellowish color, and Islam et al. (2006) whitish to light brown color to indicate high quality.

**TABLE 4:** Organoleptic characteristics of traditional and improved Bombay duck (*Harpadon nehereus*) dried fish during different storage time

Organoleptic characteristics	Initial time (0 days)		Storage time (60 days)			
	Traditional	Improved	Traditional		Improved	
			HDPE Packaged	LDPE packaged	HDPE Packaged	LDPE Packaged
Color	Blackish inner & dark brown outside	Whitish & shiny	Blackish inner & dark brown outside	Blackish inner & dark brown outside	Off white to yellowish	Yellowish to light brownish
Odor	Moderate dried fishy	Natural dried fishy	Moderate dried fishy	Slight decomposed	Natural dried fishy	Bland
Texture	Firm, tender & hard	Firm, tender & flexible	Slight soft	Moderate soft	Firm, tender & flexible	Firm, tender & flexible
Flavor	Moderate	Natural salty	Moderate	Strong	Natural salty	Natural salty
Insect Infestation	No insect infestation	No insect infestation	No insect infestation	No insect infestation	No insect infestation	No insect infestation
General appearance	Moderate good	Excellent	Moderate good	Bad	Excellent	Good
Over all acceptability	Moderately acceptable	Highly acceptable	Moderately acceptable	Slightly unacceptable	Highly acceptable	Highly acceptable
Defect point	2.42	1	2.71	3	1.28	1.57
Grade	B	A	B	B	A	A
Grade characteristics	Good/acceptable	Excellent, Highly acceptable	Good/acceptable	Good/acceptable	Excellent, Highly acceptable	Excellent, Highly acceptable

However, after 60 days storage time, moderate‐to‐severe deterioration in organoleptic characteristics were observed in terms of color (dark brown), texture (slight to moderately soft), odor, flavor, and general appearance for both products (Table 4, Figure 2). No insect infestation was found in dried fish under any production method, packaging materials, and storage time used. After storage (at days 60) in HDPE packaged conditions, the traditional dried fish was rated as “moderately acceptable” by the panelists. The HDPE packaged, improved dried fish after storage for the same time (60 days) was still rated to be as “highly acceptable” as at initial storage (Day 0). On the other hand, LDPE packaged traditional dried fish after 60 days storage time had a slightly decomposed appearance, considered “unacceptable” by panelists. LDPE packaged improved dried fish after 60 days storage time had still good appearance considered “highly acceptable.” Irrespective of packaging condition, excellent and highly acceptable dried fish was obtained from UC Davis solar chimney dryer as low‐cost improved method. During the storage period, maintenance of sensory quality of dried fish was more highly influenced by production method (traditional versus improved) than by packaging materials (HDPE versus LDPE) used. Different packaging materials, that is HDPE and LDPE, showed similar influence on the sensory quality of both traditional and improved dried fish (Figure 2).

The dried fish resulting from improved solar dryer processing at Day 0 and 60 days storage under HDPE and LDPE packaging method in the present study (Table 4) had similar color characteristics. Huque et al. (2013) and Islam et al. (2006) reported that quality dried fish products have a firm and flexible texture, have a natural odor and no insect infestation or broken pieces are detected. Those characteristics are consistent with odor, texture, and no insect infestation found in improved processed and packaged dried fish in this study. In addition, improved dried fish (both HDPE and LDPE packaged) was still highly acceptable after storage time. Likewise, Islam et al. (2006) reported quality dried marine fish remain good after three months storage. However, deterioration in color, texture, odor, and overall acceptability occurred in most dried fish samples after 5 to 7 months storage time (Islam et al., 2006). In the present study, traditionally produced dried fish were found to have deteriorated beyond acceptable quality already after 2 months. The results of this study thus indicate that solar dryer improved processed and HDPE packaged dried fish exhibits better sensorial quality over traditionally produced and LDPE packaged products after 60 days of storage time.

### 3.3 Bacteriological study of traditional and improved dried fish

In the present study, the APC, TSC, TEC, and TViC of traditional and improved dried fish under different packaging conditions and storage time are presented (Table 5). At the initial storage time (at Day 0), the APC, TSC, TEC, and TViC in traditional dried fish were 7.72 × 10^7^ CFU/g, 5.0 × 10^4^ CFU/g, 4.8 × 10^5^ CFU/g, and 3.9×103 CFU/g, respectively, which exceeds the permissible limit (lCMSF, 1986). The higher APC, TSC, TEC, and TViC in traditionally produced dried fish might be due to poor hygienic sanitary standards and condition, improper handling, and low quality of raw materials used. In the traditional process, cross‐contamination and unhygienic circumstances used for drying, insufficient use of salt, and insufficient drying time may result in poor quality dried fish. In comparison, prior to storage (at Day 0), UC Davis solar chimney dryer produced improved dried fish contained APC and TViC at levels of 4.32 × 10^4^ CFU/g and 1.3 × 10^2^ CFU/g, respectively, were within the permissible limit for dried fish (lCMSF, 1986). TEC and TSC were absent/too few to count (TFTC). The permissible limit of TSC for cooked or dried fish is 1x10^5^ CFU/g at 37°C (Surendran et al., 2006). Good quality dried fish should be totally free from *Salmonella* spp (IS, 2001). The APC, TSC, TEC, and TViC counts in improved dried fish were thus all kept within acceptable limits due to proper hygiene and sanitation practiced during handling and preparation of dried fish using the UC Davis solar chimney dryer. The result of the present study was similar with the study of Patterson and Ranjitha (2009) who found higher *E*. *coli* counts in commercially sun‐dried fish than experimental sun‐dried fish. The bacterial loads found in the current study were similar to the study of Huque et al. (2013) who found lower APC in improved solar tunnel dryer produced dried Bombay duck fish than in traditionally sun‐dried fish. Pravakar et al. (2013) reported much lower bacterial loads, at 3 × 104 cfu/g, in traditionally produced sun‐dried bombay duck fish than those found in traditional dried fish in the present study. This variation in result might occur due to variation in location, processing/preprocessing, and personnel hygiene involved between the studies.

**TABLE 5:** Microbiological characteristic of traditional and improved Bombay duck (*Harpadon nehereus*) dried fish during different storage time

Microbial parameter	Initial time (0 days)		Storage time (60 days)				Permeable load in dried fish (cfu/gm)
	Traditional	Improved	Traditional		Improved		
			HDPE Packaged	LDPE packaged	HDPE Packaged	LDPE packaged	
APC	7.72 × 10^7^±1.59	4.32 × 10^4^±1.07	3.9 × 10^5^±0.60	5.6 × 10^6^±1.30	2.3 × 10^3^±0.84	4.8 × 10^4^±0.30	≤10^5^
TSC	5.0 × 10^4^±1.10	TFTC	3.0 × 10^3^±0.90	4.7 × 10^4^±1.27	TFTC	0.5 × 10^1^±0.30	0
TEC	4.8 × 10^5^±1.2	TFTC	2.8 × 10^3^±0.29	3.6 × 10^4^±0.60	TFTC	1.0 × 10^2^±0.01	≤ 500
TViC	3.9 × 10^3^±0.40	1.3 × 10^2^±0.30	1.8 × 10^3^±0.20	2.7 × 10^4^±0.45	TFTC	0.9 × 10^2^±0.10	≤10^2^

Note

APC, Aerobic plate count; TFTC, Too few to count; TEC, Total *E.coli* count; TSC, Total *Salmonella* count; TViC, Total *Vibrio* count.

Mean ± *SD* (*n* = 3); ICMSF (1986).

After storage time (at Day 60), traditionally dried fish packaged in HDPE conditions had APC, TSC, TEC, and TViC values of 3.9 × 10^5^ CFU/g, 3.0 × 10^3^ CFU/g, 2.8 × 10^3^ CFU/g, and 1.8 × 10^3^ CFU/g, respectively, which were considerably lower than the value at Day 0. The higher oxygen barrier capability of HDPE packaging materials might result in lower microbial growth in the respective system. However, at the same 60 days storage time, traditionally dried fish samples packaged in LDPE showed higher APC 5.6 × 10^6^ CFU/g, TSC 4.7 × 10^4^ CFU/g, TEC 3.6 × 10^4^ CFU/g, and TViC2.7 × 10^4^ CFU/g values than those packaged in HDPE packaged conditions. In comparison, the APC in HDPE packaged improved dried fish was 2.3 × 10^3^ CFU/g, where no TSC, TEC, and TViC were observed. LDPE packaged improved dried fish had APC, TSC, TEC, and TViC at 4.8 × 10^4^ CFU/g, 0.5 × 10^1^ CFU/g, 1.0 × 10^2^ CFU/g, and 0.9 × 10^2^ CFU/g, respectively, which were considerably higher than in HDPE packaged samples. Both traditional and improved dried fish had lower APC, TSC, TEC, and TViC under HDPE packed conditions than under LDPE packaged conditions. The higher thickness and sterile condition of HDPE provides a higher oxygen and moisture barrier that inhibits growth of aerobic bacteria, resulting in lower bacterial count in the HDPE packaged product than in the LDPE sample. Todd (2003) reported that HDPE packages protect against dust, insects, bacteria, and germs and also provide a barrier against moisture and oxygen. Relekar et al. (2014) observed no total Coliform organisms in solar tent dried improved ribbon fish at initial stage and during entire 120 days storage, while total Coliform organisms were detected in dried ribbon fish samples collected from a local market. In the current study, initially Coliform group TEC was detected in traditional dried fish samples, however, lower than permissible limit and too few to count (TFTC) TSC, TEC, and TVC were observed in chimney dryer produced improved sample and at HDPE storage condition. Huque et al. (2013) and Das et al. (2018) also reported that dried fish without Coliform and *Salmonella* indicated acceptable quality standards. At any of these conditions, improved dried fish had bacterial loads within permissible limits where traditionally produced dried fish exceeded permissible limits. The results indicate that HDPE packaged dried fish produced with the improved method had longer shelf life than the traditional and LDPE packaged samples. Differences in physical (thickness) and barrier (moisture and oxygen) properties of packaging material resulted in better performance of HDPE over LDPE. The use of improved packaging materials further reduced the chances of microbial contamination and growth, resulting in longer shelf life of the dried fish product.

### 3.4 Biochemical analysis of dried fish sample

The biochemical quality of traditional and improved dried bombay duck (*Harpadon nehereus*) fish under different packaging conditions during storage time is presented in Table 6. Initially (at Day 0), the freshly produced improved dried fish had significantly higher protein content (59 ± 0.49%) than traditionally produced dried fish (54.46 ± 0.31%) (*p* < .05). Protein content of the same improved dried product was significantly different (*p* < .05) under different packaging HDPE (58.46 ± 0.14) and LDPE (56.97 ± 0.31) conditions at day 60 storage period, which was significantly higher (*p* < .05) than in traditional dried fish at the same condition. Significantly different protein contents were also found in HDPE and LDPE packaged traditional dried fish, at 53.56 ± 0.33% and 52.45 ± 0.51%, respectively (*p* < .05). During storage, protein content significantly (*p* < .05) reduced under any packaging condition for both traditional and improved dried products. This suggests that protein content in both traditionally and improved produced dried fish undergoes loss and decomposition during preparation and storage under any packaged condition. In this study, the protein content of *H. nehereus* dried under different conditions was found to be similar to the protein values reported in the studies by Siddique and Aktar (2011), who reported protein content in the range of 55.73%–58.33% during 6 month storage period and 53.91%–56.33% in salted sun‐dried fish reported by Bhattacharya et al. (2016).

**TABLE 6:** Biochemical characteristic of traditional and improved dried Bombay duck (*Harpadon nehereus*) fish during different storage time

Biochemical characteristics (%)	Initial time (0 day)		Traditional		Improved	
	Traditional	Improved	HDPE Packaged	LDPE Packaged	HDPE Packaged	LDPE Packaged
Protein	54.46 ± 0.31^d^	59 ± 0.49^a^	53.56 ± 0.33^e^	52.45 ± 0.51^f^	58.46 ± 0.14^b^	56.97 ± 0.31^c^
Lipid	6.00±1^a^	5.86 ± 1.12^a^	5.79 ± 1.11^a^	5.85 ± 1.1^a^	5.25 ± 0.83^a^	5.50 ± 1.11^a^
Ash	21.29 ± 0.99^a^	20.95 ± 1.30^a^	20.39 ± 0.94^a^	19.89 ± 1.19^a^	20.65 ± 1.07^a^	20.29 ± 0.94^a^
Moisture	18.24 ± 0.25^c^	15.18 ± 0.32^e^	20.25 ± 0.15^b^	21.3 ± 0.41^a^	16.63 ± 0.21^d^	18.23 ± 0.32^c^

Note

Mean ± *SD* (*n* = 3); Different alphabet in the same row indicate significant difference (*p* < .05) among different drying methods, packaging materials, and storage time.

In the current study, lipid content of dried *H. nehereus* fish ranged from 5.25% to 6.00% produced under different processing methods, packaging condition, and storage time. The result supported the study by Siddique and Aktar (2011) who reported the lipid content in dried *Harpodon nehereus* varied from 7.73% to 7.78% during 6 month storage period, 6.08 to 8.62% in solar dried fish sample (Islam et al., 2006), and 5.94 to 11.77% in salted sun‐dried products (Bhattacharya et al., 2016).

Likewise, ash content in dried *H*. *nehereus* was not significantly different among samples in this study. At the initial time (Day 0) to Day 60 storage time, ash content was in the range of 19.89 to 21.29% in traditional, improved, HDPE packaged, LDPE packaged dried fish (*p* > .05). Slight variations in ash content might result from variation in inorganic components in dried fish prepared under two different methods. Traditional fish drying is conducted in the open air which allows settling of wind‐borne dust and insect infestation. This might increase the load of higher inorganic substances, and as a consequence result in the higher ash content in the dried product. In the improved fish drying process using UC Davis solar chimney dryer, the fish are placed on trays covered with polythene, which reduces contact of dried fish with any foreign particles. This might result in lower contamination with inorganic substances. The ash content in the present study was similar with the ash content reported by Pravakar et al. (2013) and Bhattacharya et al. (2016) in sun‐dried *Harpodon nehereus* at 20.06 ± 0.36% and 14.78 to 21.96%, respectively. The packaging materials HDPE and LDPE showed similar impact on both lipid and ash content of dried bombay duck during storage period (*p* > .05). Differences in lipid and ash values among the studies might be due to differences in sampling, and processing method resulting in variation in lipid and ash content in dried bombay duck from the respective studies.

In case of moisture content, at Day 0, traditionally produced dried fish had significantly higher moisture content (18.24 ± 0.25%) than improved UC Davis chimney dryer produced dried fish (15.18 ± 0.32%) (*p* < .05). After storage time (Day 60), the moisture content (18.24 ± 0.25%) in traditional dried fish had significantly increased to 20.25 ± 0.15% and 21.3 ± 0.41% for samples packaged in HDPE and LDPE, respectively (*p* < .05), which in turn was significantly higher (*p* < .05) than the moisture content in improved dried fish (16.63 ± 0.21% and 18.23 ± 0.32% packaged in HDPE and LDPE, respectively) after the same 60 days storage period. Moisture content in improved dried fish had also increased significantly (*p* < .05) after 60 days of storage from Day 0 (15.18 ± 0.32%). The higher moisture content in the traditional product indicates that fish was not dried properly, which leads to faster deterioration of the product. Moreover, in the traditional process, dried fish processors allow incomplete drying resulting in more moisture in dried fish to gain more weight for economic benefit. For both traditional and improved produced products, HDPE packaged products had lower moisture content than LDPE packaged products (*p* < .05), indicating that HDPE packaging provides a higher barrier for water absorbability than LDPE package, which was supported by Todd (2003) that HDPE restrict oxygen and moisture in a better way than LDPE. Pravakar et al. (2013) reported 27.19% moisture content in traditionally produced dried bombay duck fish from Cox's Bazar which is similar to the current study. Other studies reported moisture contents between 11.8% and 15.0% in solar dried bombay duck fish (Islam et al., 2006) and 19.31 ± 0.48% in un‐irradiated bombay duck (Huque et al., 2013). Differences in moisture content exhibited in the current study might be due to dipping of fish into 5%–10% NaCl salt solution (in improved method) to avoid insect infestation in comparison to 10%–25% NaCl solution practiced in traditional method to get a higher weight benefit. The hygroscopic characteristics of NaCl salt bind with water in its structure (Bermeo et al., 2020), which results in a comparatively higher moisture content in the dried fish produced in that way. Irrespective of processing and packaging method, increasing moisture content was correlated with decreasing protein content during storage. Das et al. (2018) reported a similar finding that biochemical quality deteriorated for dried fish with increased relative humidity and moisture content of the products.

## 4 CONCLUSION

This paper delivers proof of concept of the UC Davis solar chimney dryer that provides an improved low‐cost method for production of premium quality dried fish. Organoleptically, the UC Davis solar chimney dryer produced improved dried fish was rated as “excellent” by panelists and considered more highly acceptable than traditionally processed dried fish both at initial (Day 0) and after 60 days storage time. The dried fish in HDPE packaging had longer shelf life and retained better sensory, microbial, and biochemical quality compared to LDPE packaged samples. The APC, TSC, TEC, and TViC of UC Davis solar chimney dryer produced improved dried fish were within permissible limits, and no *Salmonella spp*. was found both at initial (Day 0) and after 60 days of storage, while the collected traditionally processed dried fish exceeded the ICMSF permissible limit. Between two packaging conditions, the dried fish in HDPE packaged samples had better microbial quality compared to LDPE packaged samples. Nutritionally at initial time, the UC Davis solar chimney dryer produced improved dried fish had higher nutritional value compared to traditionally processed dried fish. After 60 days storage time, the nutritional quality remained good, confirming lower deterioration and longer shelf life in HDPE packaged compared to LDPE packaged conditions. Thus, quality dried fish can be produced using UC Davis solar chimney dryer in combination with improved packaging for extended shelf life that could deliver premium quality dried fish with desirable nutritional properties at acceptable domestic and international market standards.

## CONFLICT OF INTEREST

The authors declare there is no conflict of interest.

## AUTHOR CONTRIBUTIONS

**Biplob Dey Mithun:** dataCuration (lead); formalAnalysis (equal); investigation (lead); writingOriginalDraft (supporting). **Md. Sazedul Hoque:** conceptualization (lead); formalAnalysis (equal); fundingAcquisition (equal); methodology (lead); projectAdministration (lead); supervision (lead); validation (equal); visualization (lead); writingOriginalDraft (equal); writingReviewEditing (lead). **Md. Mahmudul Hasan:** dataCuration (supporting); formalAnalysis (equal); investigation (supporting); methodology (equal); resources (equal); writingOriginalDraft (supporting). **Shaida Akter:** dataCuration (supporting); formalAnalysis (equal); investigation (supporting); resources (supporting); writingReviewEditing(supporting). **Mohd. Rezaul Islam:** conceptualization (supporting); methodology (supporting); projectAdministration (equal); resources (equal); validation (supporting); writingReviewEditing (supporting). **Martin Louis van Brakel:** conceptualization (supporting); methodology (supporting); supervision (equal); validation (equal); writing Review Editing (equal).

## ETHICAL REVIEW

The study involved the human for sensory quality analysis of dried fish which was conducted following the ethics approval from the Research and Training Center of Patuakhali Science and Technology University, Bangladesh.

## INFORMED CONSENT

Written informed consent was obtained from all study participants.

## Data Availability

Research data are not shared.
